# Translation of Medical AR Research into Clinical Practice

**DOI:** 10.3390/jimaging9020044

**Published:** 2023-02-14

**Authors:** Matthias Seibold, José Miguel Spirig, Hooman Esfandiari, Mazda Farshad, Philipp Fürnstahl

**Affiliations:** 1 Research in Orthopedic Computer Science, Balgrist University Hospital, University of Zurich, CH-8008 Zurich, Switzerland; 2 Computer Aided Medical Procedures and Augmented Reality, Technical University Munich, DE-85748 Garching, Germany; 3 Department of Orthopedics, Balgrist University Hospital, University of Zurich, CH-8008 Zurich, Switzerland

**Keywords:** translational research, medical AR, orthopedic surgery, pedicle screw placement, computer aided surgery

## Abstract

Translational research is aimed at turning discoveries from basic science into results that advance patient treatment. The translation of technical solutions into clinical use is a complex, iterative process that involves different stages of design, development, and validation, such as the identification of unmet clinical needs, technical conception, development, verification and validation, regulatory matters, and ethics. For this reason, many promising technical developments at the interface of technology, informatics, and medicine remain research prototypes without finding their way into clinical practice. Augmented reality is a technology that is now making its breakthrough into patient care, even though it has been available for decades. In this work, we explain the translational process for Medical AR devices and present associated challenges and opportunities. To the best knowledge of the authors, this concept paper is the first to present a guideline for the translation of medical AR research into clinical practice.

## 1. Introduction and Related Work

Digitization, as part of technological transformation, will have a sustainable impact on the development of the healthcare sector in the years ahead. In fact, technology will be a key component for addressing the major global healthcare challenges posed by an aging society. The key factors for this development are the automatic large-scale collection of health-related data, the application of data-driven methods to generate knowledge from this data, and the extraction of treatment-relevant information as well as the enhancement of the capabilities of humans by new technologies, such as artificial intelligence (AI), augmented reality (AR), and robotics.

One of the key emerging technologies driving digitalization is AR, which has gained increasing popularity in the medical domain in recent years [1]. AR has great potential to improve surgical treatments and provide information to surgeons in an intuitive way. Nonetheless, AR is also associated with a high degree of technological complexity. One of the major advantages of using AR in surgery are intuitive and context-aware visualization capabilities, which allow for overlay computer-generated 3D information, such as medical imaging data or information from preoperative planning for surgical guidance onto the real patient anatomy. AR can also enable sterile interaction through gesture- and voice-controlled 3D interfaces, reducing the operator’s mental load in the highly challenging OR environment. In recent years, new AR head-mounted displays (HMDs) have been developed that are capable of localizing their position within the environment using device-embedded sensors and algorithms such as simultaneous localization and mapping (SLAM). These achievements paved the way for enabling the application of AR-HMDs in the medical domain. Furthermore, embedded sensors for tracking the operator’s eyes and hands, as well as capturing an egocentric view of the surgical scene through device-embedded cameras, provide rich information about the surgical context, which can be exploited for a variety of applications in computer assisted surgery and surgical guidance [2]. The usage of AR for intraoperative visualization has been described in the literature for rigid anatomies such as bone [3] and for soft-tissues structures [4] such as nerves and the spinal cord. First commercial AR systems have received FDA clearance such as the xvision system by Augmedics (Arlington Heights, IL, USA) (https://augmedics.com/, accessed on 14 December 2022) or the NextAR platform by Medacta (Castel San Pietro, Switzerland) (https://nextar.medacta.com/, accessed on 14 December 2022). However, many research projects do not make the step from a research prototype into a successful commercial product and have been mostly developed and validated in controlled environments on phantom or human cadaveric specimens [3,5]. The objective of this work is to provide a guideline for translational research in the field of medical AR, which we believe to be of high interest for the research community. In the following sections, we showcase the necessary steps and related challenges in each development stage of a medical AR product. Our goal here is to provide a summary of the associated challenges and our own learnings including not only the technical development process, but also regulatory, ethical, and commercial considerations. We illustrate the concept of a translational research process in the context of a previous research project conducted within our research group.

**Figure 1:**
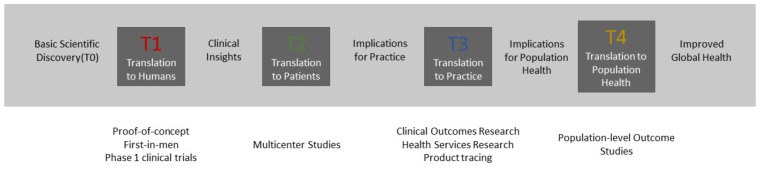
The process of translational research from basic scientific discovery to translation to population health [6].

Clinical translation includes a spectrum of activities and interactions between various disciplines and stakeholders that can be categorized into four large phases (T1–T4) [6], as illustrated in Figure 1. Phases T0–T2 aim to translate a fundamental scientific discovery into proof-of-concepts, extend them to medical devices, evaluate their performance in clinical trials, and introduce them into clinical practice. Phases T3–T4 cover long-term clinical outcome research and population-level outcome studies that have the goal of generating clinical insights and investigating the implications for practice and population health. Research and Development (R&D) activities in the context of medical AR mainly happen within phases T0 and T2. We propose dividing the translational process of these phases into different development stages using technology readiness levels (TRL). TRL is a measurement system that was originally developed the National Aeronautics and Space Administration (NASA, Washington, DC, USA) to assess the maturity level of space technology^1^. This scale has already been proven to be suitable for application in the medical device development realm [7] and for the maturity assessment of medical AR systems in a literature review conducted by Eckert et al. [1]. The completion of each TRL stage requires validation of increasing complexity to ensure that the requirements and specifications are met before moving to the next stage. Table 1 provides an overview of our proposed TRL schema adapted from the original NASA scale.

## 2. Incremental Research and Development towards Translation from Phase T0 to T2

In the following sections, we showcase the translational process of medical AR research from an early-stage proof-of-concept towards clinical studies, explained through an exemplary use case from spinal fusion surgery called *HoloNavigation* [8,9,10,11,12,13]. The *HoloNavigation* project was conducted within a large research initiative called SURGENT (https://www.hochschulmedizin.uzh.ch/en/projekte/surgent.html, accessed on 7 September 2022), an acronym for Surgeon Enhancing Technologies. SURGENT is an ambitious multi-institutional and inter-disciplinary project with the goal of bringing medical AR innovation into real surgeries. One product resulting from *HoloNavigation* consists of two AR-based surgical navigation applications to assist surgeons during pedicle screw placement and the adaption of spinal rod implants. The key idea of the AR-based navigation of pedicle screw placement is to determine the optimal screw insertion points and trajectories through 3D preoperative planning, to transfer the planning to the intraoperative anatomy through surface digitization-based registration, and to display insertion points and screw trajectories directly in situ. Navigation of rod implant bending relies on the visualization of a virtual model of the optimal rod shape, which is determined from an AI-based 3D reconstruction of the pedicle screw head positions. The adaptation of a rod implant to the patient’s anatomy is then determined by using the virtual rod model as a visual template for bending the real implant.

**Figure 2:**
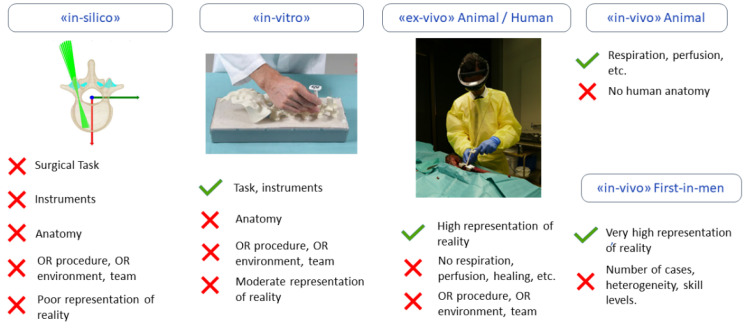
Validation models that are utilized in the different development stages of medical AR systems. For each validation model the included (✓) and excluded (✕) validation capabilities and components are indicated below each model, respectively. In the translational research process of medical AR systems, “in silico” and “in vitro” validation models are typically used in TRL3, “ex-vivo” animal studies are usually applied in TRL4, “ex vivo human” and “in vivo” animal experiments are performed in TRL5, and an “in vivo first in-man” study is performed in TRL6, as described in the following paragraphs.

In the following sections, we apply the TRL scheme to categorize medical AR R&D activities toward translation into nine incremental steps along with TRL-specific validation models (see Table 1 and Figure 2).

### 2.1. Early Concept and Research Prototype (TRL1–TRL3)

The translational research process starts with a research idea inspired by an unmet medical need (TRL1) that has been observed in practice. The scientific findings are reviewed against the current state-of-the-art and a hypothesis is formulated. The basic principle is then reported in a research proposal and a project plan is created, which allows for the acquisition of project funding.

In TRL2, the research project starts with the goal of demonstrating key performance features of the novel system. Hereby, a scientific team consisting of PhD students, senior researchers, and clinicians are tasked with the design and development of a research prototype. This process is usually associated with lower costs in comparison with corporate research. The projects start with a three-step process where clinical requirements, system components, and technical specifications are elaborated on through a process called clinical requirement engineering. Clinical requirement engineering defines a standardized and formal representation of a treatment through process models. In the case of *HoloNavigation*, a process model is created for the use case of spinal fusion surgery [8] by hierarchically decomposing the operation into phases, steps, sub-steps, and actions [14,15].

After the system components and their technical specifications have been derived from the clinical requirements, the first research prototypes are developed through an incremental prototyping process (TRL 3). While the goal is to demonstrate the key performance features as part of a proof of concept, regulatory, ethical, and commercial considerations should also be incorporated from the onset. These include special requirements for software validation (e.g., AI software) and hardware sterilization, as well as considerations for integration into the OR and the surgical workflow. In the case of our project, hardware prototypes of 3D printed surgical instruments as well as software for anatomy registration, instrument localization, and surgical guidance were developed, deployed to the Microsoft HoloLens, and tested. Proof-of-concepts were incrementally extended and verified in a limited number of laboratory experiments. In the early development stages, computer simulations (“in silico”) and first experiments using synthetic anatomy models (“in vitro”) were conducted as illustrated in Figure 2.

In these early stages of development, coordination and alignment with all stakeholders is critical to ensure a close collaboration between the technical and medical experts to successfully address the clinical needs.

### 2.2. Integrated Prototype Development (TRL4–TRL5)

In TRL4, an integrated system prototype is developed and validated that contains all components of the envisioned final product. This development includes integration activities and systemwide performance optimizations towards the usage of the prototype in pre-clinical studies. The intended specifications of the prototype are validated by surgeons as intended users in final in vitro experiments. Besides key performance, additional factors such as potential safety problems or adverse events are recorded. To this end, TRL4 prototypes should be validated in the real environment (e.g., OR), where, for instance, special illumination conditions with surgical lights could hinder the proper function of certain components. Another considerable factor is the potentially limited working ergonomics of physicians while wearing the HMD [16].

In the TRL4 study of *HoloNavigation*, 160 drill pilot holes for pedicle screws were placed by four surgeons in synthetic vertebra models (Synbone, Zizers, Switzerland) which were embedded in agar-based gel to better simulate a realistic surgical exposure [17]. After the pedicle screw placement was navigated, AR-guided rod implant bending was performed [13]. The primary outcome measure for pedicle screw placement was defined as the accuracy of the surgical execution quantified by comparing the screw entry points and trajectories with the optimal values from the preoperative plan. The rod bending performance was assessed using re-bending attempts and bending time. The study proved that the bending time can be reduced by up to 20% compared with the current clinical gold standard. The performance of the integrated prototype in terms of the clinical requirements were frequently discussed with surgeons during and after the experiments. Insufficiently fulfilled requirements such as inaccurate superimposition of the holographic projection due to inaccurate user registration or an insufficient holographic visualization were identified and improved. Hereby, it is important to note that a large part of the incremental refinement can take place within TRL4 and in the laboratory. An exception to the aforementioned points is AI methods, where the realistic anatomy should be included as early as possible as their performance is determined almost entirely by the underlying data. 

Once all of the requirements and specifications are met, a code freeze is performed before validating an integrated prototype in the relevant environment (TRL5), which involves more elaborate and costly validation on ex vivo human and/or in vivo animal models. These models represent the current pre-clinical gold standard of validation, providing anatomical, biomechanical, and physiological features that closely resemble those of living humans. In particular, the influence of soft tissues, body fluids, or respiration can be investigated more effectively. Ex vivo human and in vivo animal validation requires the experiments to be approved by a responsible ethical committee according to the human research act (https://www.bag.admin.ch/bag/en/home/gesetze-und-bewilligungen/gesetzgebung/gesetzgebung-mensch-gesundheit/gesetzgebung-forschung-am-menschen.html, accessed on 21 October 2022) or the animal welfare act (https://www.globalanimallaw.org/database/index.html, accessed on 14 December 2022), respectively. In the *HoloNavigation* project, TRL5 performance was validated using two cadaveric lumbar spine specimens, which were placed in a prone position to mimic a real surgical case [12]. A common surgical approach was performed through a midline incision followed by creating a sufficient bone exposure. After registration of the bony anatomy by surface digitization [11], k-wires were placed according to the holographic projection of the planned screw trajectories. The results of this study showed improved adherence to the preoperatively planned trajectory angle (5.88 ± 3.69°) when compared with the freehand technique (11.21 ± 7.64°). However, hardware instability remained, and key performance features such as surface digitization and registration were difficult to apply due to the soft tissue coverage of the bone and the deep surgical situs. 

A major rehaul was conducted by switching the hardware to the next generation of the Microsoft HoloLens, redesigning markers and instruments, and modifying software to improve the robustness of the registration. A follow up validation study on cadavers was launched [10], which confirms the iterative character of the research and development as mentioned above. This second study included eight cadavers and two experienced surgeons, as well as two biomedical engineers as laymen operators. The study results proved the safety and operator independent reliability of the system.

### 2.3. Clinical Evaluation (TRL 6–TRL7)

The results and documentation of pre-clinical studies lay the groundwork for conducting clinical trials to assess the safety, efficacy, and risks associated with the use of the final or near-final device with patients. In most cases, these trials are led by an industrial commercialization partner that intends to introduce the medical product to the market. The commercialization partner develops the necessary documentation for regulatory and ethical approval, implements the study in appropriate centers, and covers associated costs. In the case of medical AR products, compliance to the medical device regulations (FDA (https://www.fda.gov/, accessed on 21 October 2022) and MDR (https://www.swissmedic.ch/swissmedic/de/home/medizinprodukte/regulierung-medizinprodukte/neue-eu-verordnungen-mdr-ivdr.html, accessed on 21 October 2022)) and medical device standards for, e.g., software, safety and ergonomics, usability, risk management and quality management, and cleaning/sterilization, must be documented. A useful resource in this context is a paper about AR and VR for medical devices published by the FDA (https://www.fda.gov/media/159709/download, accessed on 14 December 2022).

Clinical trials are broken up into a series of phases, each with a specific objective. Phase 1 studies or first-in-man studies assess the safety of a new medical device during its first use on real patients (TRL6). This phase requires a highly standardized and controlled environment compared with routine treatments such as a small and homogeneous study population or additional safety measures such as additional fluoroscopy-guided control steps. In the first-in-man study of the *HoloNavigation* project, three single-level spinal fusion surgeries were performed by two experienced spine surgeons [9]. The highly dynamic environment of a real surgery with an interacting OR team revealed new limitations of the device with respect to the SLAM tracking performance, voice command recognition, and user interface. The necessary modifications made system improvements and ex vivo re-testing necessary before moving on to the next phase of the clinical trial.

The purpose of a phase 2 study (TRL7) is to not only assess the safety, but also efficacy of the system. Efficacy can be defined as the performance of an intervention under controlled circumstances, which usually requires a study design in the form of a randomized controlled trial (RCT). RCTs compare the efficacy of a new medical product with that of an established gold standard by assigning patients randomly to either the interventional or control group, respectively. The number of patients included must be sufficiently large to allow for systematic conclusions based on statistical testing. In the *HoloNavigation* project, an RCT study with 60 patients had been prepared and submitted for approval to the regulatory authorities.

### 2.4. Implementation into Practice (TRL8–TRL9)

Depending on the risk assessment, medical devices may still need to undergo larger studies to confirm their clinical effectiveness, which are referred to as phase 3 studies (TRL8). Clinical effectiveness assesses the performance of the device in routine treatment and under real-world conditions. To this end, multicenter studies are being conducted with a large heterogeneous patient population from different hospitals located in several countries and operated by surgeons with different educational backgrounds and skill levels.

The final stage of the proposed scale is TRL9, which is achieved once the device is introduced by the industrial partner into the global market after regulatory approval. After TRL9, the long-term effectiveness in the general population is continuously monitored through post-market surveillance and phase 4 studies.

## 3. Challenges and Opportunities

The era of traditional computer-assisted surgery is ending without ever having become the standard of care. Emerging data- and sensor-driven technologies, most notably robotics, AI, and AR, enable a holistic approach where all available data can be used to make treatments safer, more precise, and more standardized [18]. These developments have enormous potential to advance medicine in a sustainable way. However, they also pose new challenges for science and research.

### 3.1. Technical Challenges

One of the major technical challenges in the medical, and especially the surgical domain, is the acquisition, storage, annotation, standardization, and exchange of large amounts of data, which are fundamentally needed for the implementation of data-driven approaches [19]. This data can theoretically be derived from patients, surgeons, OR devices, and the OR team. However, in real treatment, only a fraction of these data are digitized and stored in a structured way due to proprietary interfaces, regulatory constraints, workflow limitations, or safety considerations. Conversely, the state-of-the-art in research relies on extensive imaging and computer vision setups for data collection that are not approved for OR usage. Benefits for patients as well as costs for regulatory and implementation do not yet justify the integration of such setups into routine treatment.

Therefore, lack of sufficient data is a major bottleneck that limits the capabilities and generalization of methods and, consequently, hinders their translation and distribution into clinical practice. Worldwide, endeavors are made to improve the current situation by establishing new infrastructures, creating alliances and initiatives, and developing new research approaches, e.g., the automatic annotation of ground truth data [20] or the usage of large synthetic datasets for model pretraining [2]. On an infrastructure level, technically highly equipped simulation centers such as the SESAM human simulation center (https://www.sesam-web.org/centres/centre/human-simulation-center-hsc/, accessed on 21 October 2022) in Germany, the SIE Validation Suite (https://www.kcl.ac.uk/bmeis/our-departments/surgical-interventional-engineering, accessed on 21 October 2022) in the UK, or the OR-X in Switzerland, enable the simulation of interventions and entire surgeries under realistic conditions to accelerate and facilitate data collection, technology integration, and validation. Equally important is the development of data standards and governance policies which are addressed by national and international initiatives such as the LOOP Zurich (https://www.theloopzurich.ch/de/, accessed on 21 October 2022), the surgical data science initiative (http://www.surgical-data-science.org/, accessed on 21 October 2022), or the EU’s Open Science policy (https://research-and-innovation.ec.europa.eu/strategy/strategy-2020-2024/our-digital-future/open-science_en, accessed on 21 October 2022). 

A main requirement of the technologies used in medicine is accuracy and reliability (e.g., of measurements or surgical guidance visualizations), which remains a challenge for medical AR. SLAM-based inside-out tracking is a key component for the wide adoption of HMDs in the medical domain, and promising results have been reported using AR-HMD-based navigation systems in a number of experiments, e.g., for application in endovascular aortic repair [21] or pedicle screw placement [11], especially in comparison with conventional free-hand techniques without computer aid [12]. However, because of the individual challenges in the OR, inside-out tracking algorithms of consumer-grade HMDs do not reach the same level of accuracy. Even though medical-grade high-precision tracking systems provide very accurate measurements, the development of specialized self-tracking solutions incorporating the individual challenges of the operating room has great potential for the use and adoption of HMDs in the OR by removing the constraints present in outside-in tracking systems such as line-of-sight issues.

Medical AR is usually only one part of a complex system of different technologies, which together form the final medical device solution. For example, many medical AR systems are combined with AI and computer vision algorithms, or collaborate with additional hardware such as tracking systems, robots, or imaging devices [2,20,22,23]. The system complexity makes the validation of and implementation as a medical device challenging, expensive, and time-consuming. As further described in Chapter 3.2, dedicated infrastructures for testing and validation are needed to decrease development costs and bench-to-bed time. 

Another challenge of the translational process for medical AR is the incorporation of highly specialized expertise from different technical and medical domains. Close collaboration and interaction are key factors to transform clinical needs into a medical AR product for patient treatment. Frequent discussions between medical and technical professionals are necessary to constantly reiterate and reevaluate the different stages of development. Furthermore, interdisciplinary development of clinical-technical solutions requires new forms of interaction and collaboration, as the technology is evolving and entering new fields of medical practice [24].

Another challenge concerns the optimization of the regulatory pathway for complex, adaptive technologies such as medical AI or AR devices. The traditional paradigm of medical device regulation was not designed for these technologies. The first steps have to be undertaken by authorities, industry, and academia, but all parties need to gain more experience to make translation faster and more efficient. The Federal Drug Administration (FDA) described in a discussion paper (https://www.fda.gov/media/122535/download, accessed on 21 October 2022) that the highly iterative, autonomous, and adaptive nature of these technologies requires a new, total product lifecycle regulatory approach that facilitates a rapid cycle of product improvement and allows these devices to continually improve while providing effective safeguards. A software precertification program (https://www.fda.gov/media/113802/download, accessed on 21 October 2022) was called into life that embodies a voluntary regulatory model to assess the safety and effectiveness of software technologies without inhibiting patient access to these technologies. In turn, translational research also needs to form a better understanding of regulatory processes to consider regulatory requirements in early TRLs. This can be achieved by either building up dedicated units with regulatory expertise or by pairing up early with industrial partners as described in Section 3.3.

### 3.2. OR-X—A Translational Center for Surgical Excellence

New infrastructures are needed in order to provide research with early access to a realistic (OR) environment for conducing data collection, incremental prototyping, and validation experiments. A prime example of such an infrastructure is the OR-X (https://or-x.ch/, accessed on 21 October 2022) at the University Hospital Balgrist in Zurich. The OR-X is designed to create an optimal and adaptable environment for the research, development, and implementation of surgical innovations. The OR-X consists of a fully equipped surgical core facility including a research-OR and a surgical training lab, in which entire cadaveric surgeries can be performed under highly realistic conditions. OR-X research is driven by three future-oriented technological units that will tackle the major challenges of the increasing digitization of surgical care.

The three technological units of the OR-X supply specific infrastructure and personnel focusing on key areas of data-driven research and innovation in surgery. The Unit for Intraoperative Imaging (UII) combines state-of-the-art intraoperative imaging technologies with robots and new sensor technologies, allowing researchers to exploit the full potential of intraoperative imaging and to develop advanced image-based methods for intraoperative decision making and error prevention. The Unit for Surgical Data Science (USDS) is the data hub of the OR-X where all data sources are aggregated into large databases of semantic and quantitative information about surgeries. These data collection capabilities will be a key asset of the OR-X to elevate research on surgical data science. The mission of the Unit for Surgical Execution and Translation (USET) is to accelerate the translation of surgical innovations into patient treatment by providing a testbed for incremental prototyping, technical verification, and validation in a highly realistic surgical environment.

### 3.3. Exploitation Strategies

Commercialization is necessary to sustainably translate AR solutions from bench to bedside. This is particularly important in the MedTech field, as the translation to phase T1 is associated with high costs, given factors such as strict regulatory and ethical processes, challenges in market access, and highly regulated quality standards. Depending on the solution, commercialization can be achieved at different stages of the product development and through different exploitation strategies.

A common strategy is to develop the proof-of-concept prototype along with the required rudimentary validation studies in the academic environment where the initial research idea was created and the clinical requirements were established. Given the lack of in-house expertise in regulatory processes and quality management systems, research and development entities who have a promising prototype generally reach out to external MedTech companies for potential partnerships. Such partnerships can be established at different phases of the product development: (1) after initial lab validations (TRL = 4), (2) after ex vivo lab validations (TRL = 5) or (3) after in vivo first-in-human studies (TRL = 6). In general, the further the prototype is advanced in the lab setting, the easier it becomes to establish the aforementioned partnerships; however, one should factor in the expenses that arise from the conduction of the required validation studies in an academic environment in order to achieve a higher TRL. Large MedTech companies already have processes in place whereby new products or solutions can be implemented into the market through appropriate regulatory pathways. Alternatively, the entire bench-to-bedside transition process can be conducted in-house, given that the availability of the required resources for establishing the regulatory affairs and the quality management standards are available. This may be possible in small entities such as startups through the support of business incubators or external consultancy firms that specialize in medical device certification and quality management systems.

Regardless of the timing of the aforementioned translation, one should consider the market reach when deciding upon the most appropriate strategy. Medical AR solutions may have niche target markets in form of specialized healthcare providers, hospitals, or private practices. Access to this market segment can be challenging, because global costumer and distribution networks are required. Therefore, for these products, it might be a more viable option to pursue business-to-business strategies (B2B), where a licensing agreement is made with a large MedTech entity that takes the lead on the distribution and marketing of the product in exchange for business shares or revenue. In contrast, AR products that are developed for the end user or patient (e.g., rehabilitation solutions), may be directly presented and sold to the clients upon the establishment of proper distribution channels through a business-to-client (B2C) model.

Given the increasing pace of hardware and software development in the realm of medical AR, a steady and reliable customer relations process must be in place to enable product upgrades or software and firmware updates. Although this is relevant only when the product is already in the market, one should consider it as an additional criterion that may define the transition strategy of small startups. This may also be seen as a leverage that may enable small entities to maintain their market presence.

## 4. Conclusions

In this concept paper, we introduced a new guideline for the translation of a medical AR device into clinical practice, and illustrate the individual development and validation stages based on the example of the research project *HoloNavigation*. The paper should serve the community as a guideline for translational research in the strongly growing field of medical AR.

## Figures and Tables

**Table 1:** The proposed technology readiness level (TRL) scale for medical AR products was obtained by adapting the original NASA (https://www.nasa.gov/directorates/heo/scan/engineering/technology/technology_readiness_level, accessed on 7 September 2022) TRL scale.

TRL	Description
**1**	Basic principles observed and reported in a hypothesis. State-of-the-art reviewed and scientific proposal articulated.
**2**	Technology concept defined through clinical requirements, system components, and technical specifications.
**3**	Development and verification of a research prototype providing proof-of-concept of some key performance features.
**4**	Integrated prototype finalization and validation in a laboratory environment. Besides key performance, additional factors such as safety or adverse events are evaluated.
**5**	Validation of the prototype in relevant environment (ex vivo human/in vivo animal). Generation of pre-clinical data to justify in vivo studies.
**6**	Near-final device demonstrated safety in its first use on patients in a realistic, but highly controlled and standardized environment (“first-in-human” phase 1 study).
**7**	Device demonstrated safety and efficacy in a controlled operation environment through a randomized controlled trial (phase 2 study).
**8**	Clinical effectiveness, safety, and risks in using the device under real-world conditions are successfully investigated in large multi-center studies (phase 3 studies)
**9**	Regulatory approval for using the device in routine treatment obtained. Long term effectiveness and usage is monitored through post-market surveillance (phase 4 studies).

## Data Availability

Not applicable.
